# Masked Corneal Opacity in Bullous Keratopathy: The Impact of Epithelial Pathology on Surgical Decision-Making—A Case Report

**DOI:** 10.3390/jcm15114059

**Published:** 2026-05-24

**Authors:** Wojciech Luboń, Łukasz Drzyzga, Wojciech Rokicki, Dorota Wyględowska-Promieńska

**Affiliations:** 1 Department of Ophthalmology, Faculty of Medical Sciences, Medical University of Silesia, 40-514 Katowice, Poland; w.rokicki@sum.edu.pl (W.R.); dwygledowska@sum.edu.pl (D.W.-P.); 2 Department of Ophthalmology, Professor K. Gibiński University Clinical Center, Medical University of Silesia, 40-514 Katowice, Poland; drzyzgalukasz@gmail.com

**Keywords:** bullous keratopathy, corneal opacity, epithelial remodeling, epithelial debridement, endothelial keratoplasty, DSAEK, case report

## Abstract

Bullous keratopathy may lead to severe corneal opacity and impaired visualization of anterior segment structures, complicating surgical qualification for endothelial keratoplasty (EK). We report the case of a 67-year-old male with pseudophakic bullous keratopathy and Fuchs endothelial dystrophy presenting with clinically complete corneal opacity and visual acuity limited to hand motion. Slit-lamp examination and anterior segment optical coherence tomography demonstrated marked epithelial remodeling with a dense plaque-like surface lesion obscuring deeper corneal structures. A staged intraoperative approach was undertaken. Following mechanical epithelial debridement, partial restoration of corneal transparency allowed for an intraoperative reassessment of stromal clarity and subsequent Descemet Stripping Automated Endothelial Keratoplasty (DSAEK). Histopathological examination demonstrated reactive epithelial thickening with associated subepithelial fibrosis consistent with chronic bullous keratopathy. Postoperatively, corneal transparency was restored and best-corrected visual acuity improved to 0.7 Snellen (0.15 logMAR), remaining stable during follow-up without graft-related complications or recurrent epithelial abnormalities. This case highlights the importance of considering epithelial contributions to apparent corneal opacity in advanced bullous keratopathy and suggests that staged intraoperative reassessment may support individualized surgical decision-making in selected patients with inconclusive preoperative evaluation.

## 1. Introduction

Bullous keratopathy represents a final common pathway of corneal endothelial failure, leading to progressive stromal edema, epithelial bullae formation, recurrent erosions, and ultimately loss of corneal transparency. The condition most frequently develops secondary to surgical trauma, particularly following cataract extraction, as well as in the course of endothelial dystrophies or other causes of endothelial decompensation [1,2]. Chronic endothelial dysfunction results in persistent corneal hydration, which disrupts stromal architecture and induces secondary changes in the corneal epithelium, including thickening, irregularity, and impaired barrier function.

Over the past two decades, the management of endothelial failure has undergone a paradigm shift with the introduction of endothelial keratoplasty (EK), which has largely replaced penetrating keratoplasty (PK) as the preferred surgical approach. Techniques such as Descemet stripping automated endothelial keratoplasty (DSAEK) and Descemet membrane endothelial keratoplasty (DMEK) allow selective replacement of the dysfunctional endothelium while preserving the native corneal stroma, resulting in faster visual recovery, improved refractive outcomes, and lower complication rates compared with full-thickness transplantation [3,4,5,6,7]. Previous studies have reported favorable anatomical and functional outcomes following EK, including high graft survival rates and improved visual acuity [8,9,10].

Despite these advances, appropriate patient qualification for endothelial keratoplasty remains critically dependent on accurate preoperative assessment of corneal structure and visual potential. In routine clinical practice, the decision between EK and PK is primarily based on the presumed extent of stromal involvement and the degree of corneal opacity. Eyes presenting with dense, diffuse corneal opacification are often considered poor candidates for lamellar procedures and may be directed toward PK, which is associated with a higher surgical burden and increased risk of complications [5,7,11].

However, the contribution of secondary epithelial pathology to the overall loss of corneal transparency is frequently underrecognized. Chronic epithelial edema in bullous keratopathy may lead to significant morphological remodeling of the corneal surface, including epithelial hyperplasia, irregular keratinization, and the formation of dense, plaque-like structures. These changes can obscure the underlying stromal architecture and simulate full-thickness corneal opacification, thereby potentially leading to misinterpretation of surgical eligibility. Recent observations indicate that epithelial remodeling may also influence postoperative outcomes by altering corneal optics and surface regularity following endothelial keratoplasty [12].

In this context, distinguishing between stromal and epithelial components of corneal opacity may be important for optimal surgical planning. Failure to recognize reversible epithelial pathology may result in inappropriate exclusion of patients from less invasive lamellar procedures. Conversely, targeted management of the corneal surface, including mechanical epithelial debridement or superficial keratectomy, may restore sufficient transparency to enable accurate intraoperative assessment and facilitate endothelial transplantation [13].

The aim of this study is to present a case of advanced bullous keratopathy in which dense pathological epithelial remodeling completely masked the underlying corneal structure and initially suggested the need for PK. Mechanical removal of the pathological epithelium revealed preserved stromal transparency, allowing successful DSAEK and substantial visual improvement.

## 2. Case Presentation

A 67-year-old male was referred to the Department of Ophthalmology of the University Clinical Center in Katowice, Poland, due to progressive visual deterioration in the right eye. The patient reported gradual worsening of vision over approximately 10 months. Despite significant visual decline, no surgical intervention had previously been undertaken due to concerns regarding procedural risk, and the patient had initially declined operative treatment. Surgical consideration was revisited when vision deteriorated to a level significantly impairing daily functioning.

The patient’s ophthalmic history was notable for pseudophakia and coexisting Fuchs endothelial corneal dystrophy. Endothelial cell density in the affected eye could not be measured due to complete corneal opacity precluding reliable specular microscopy assessment. The fellow eye presented with reduced visual acuity of 0.4 Snellen (0.40 logMAR), associated with cataract and Fuchs dystrophy. Specular microscopy in the left eye demonstrated decreased endothelial cell density (approximately 1800 cells/mm^2^), with polymegathism and pleomorphism consistent with endothelial dysfunction.

The patient’s medical history included type 2 diabetes mellitus and moderate chronic heart failure. Based on systemic status, the perioperative risk was classified as American Society of Anesthesiologists (ASA) physical status III.

### 2.1. Functional Status Prior to Treatment

At presentation, best-corrected visual acuity (BCVA) in the right eye was limited to hand motion. The patient reported severe impairment in daily activities, including compromised spatial orientation and inability to perform tasks requiring binocular vision. The progressive deterioration of vision over recent months significantly affected quality of life and ultimately motivated the patient to reconsider surgical intervention.

In addition to visual impairment, the patient experienced persistent ocular discomfort related to advanced bullous keratopathy. He reported recurrent episodes of ocular pain, foreign body sensation, and stabbing sensations associated with epithelial bullae. These symptoms were particularly pronounced during blinking and exposure to environmental factors such as wind or light. The chronic nature of these complaints resulted in significant functional limitations, reduced tolerance for daily activities, and a marked decrease in vision-related quality of life. The chronological course of the patient’s symptoms, diagnostic assessment, treatment, and follow-up is summarized in Table 1.

### 2.2. Baseline Clinical Examination

**Figure 1:**
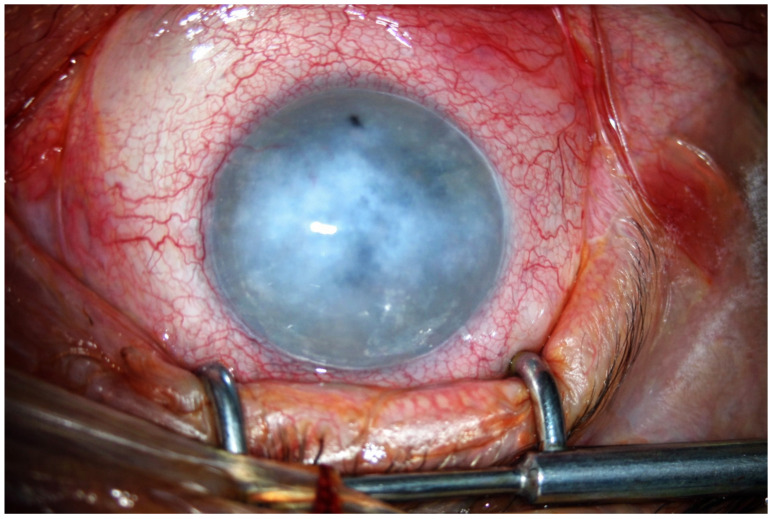
Slit-lamp image demonstrating complete corneal opacity with a dense, thickened plaque-like epithelial layer in advanced bullous keratopathy.

Slit-lamp examination of the right eye revealed clinically complete corneal opacity associated with advanced bullous keratopathy. The corneal surface was covered by a dense, irregular, thickened plaque-like epithelial lesion forming a thick, crust-like structure that entirely prevented visualization of the anterior chamber (Figure 1). The surface appeared markedly uneven, with a dull, opaque appearance and loss of normal corneal luster, consistent with severe epithelial pathology and chronic surface remodeling.

**Figure 2:**
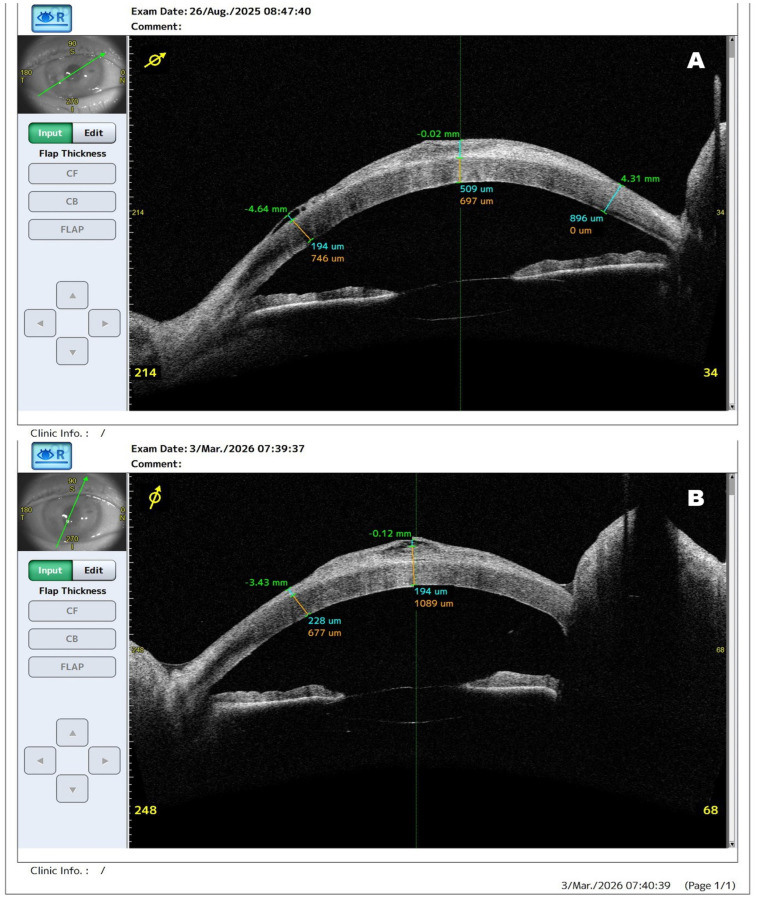
AS-OCT images of the affected eye. (**A**) Initial scan demonstrating a markedly thickened, irregular, hyperreflective epithelial layer forming a prominent plaque-like surface lesion, with obscuration of deeper corneal structures and limited visualization of the underlying stroma. (**B**) Preoperative scan confirming persistent epithelial thickening, pronounced surface irregularity, epithelial bullae, and diffuse stromal edema associated with advanced bullous keratopathy.

AS-OCT demonstrated a markedly thickened, irregular, and hyperreflective epithelial layer forming a prominent, protruding plaque over the corneal surface, consistent with advanced pathological epithelial remodeling (Figure 2A). The epithelial layer appeared heterogenous in reflectivity, with areas of increased signal intensity suggesting abnormal tissue organization and surface keratinization. The interface between the epithelium and the underlying stroma was poorly delineated, further complicating structural assessment.

Repeat imaging confirmed persistence of these abnormalities, with pronounced surface irregularity and multiple epithelial bullae corresponding to chronic epithelial edema secondary to endothelial failure (Figure 2B). The corneal surface remained highly uneven, with focal elevations and disruptions in epithelial continuity, contributing to significant optical distortion.

Due to the severity of the epithelial pathology, reliable preoperative evaluation of stromal transparency, posterior corneal layers, endothelial status, and anterior chamber details was not possible. Although AS-OCT provided partial structural information regarding the anterior corneal surface, the dense and irregular epithelial lesion substantially limited assessment of deeper corneal structures and prevented accurate preoperative determination of surgical eligibility.

Intraocular pressure (IOP) prior to surgery was 20 mmHg. Posterior segment evaluation using B-scan ultrasonography revealed no evidence of calcifications, abnormal thickening of the ocular coats, or significant degenerative changes, suggesting preserved posterior segment integrity.

### 2.3. Prior Conservative Management

Before surgical qualification, the patient had undergone prolonged conservative treatment, including topical hyperosmotic therapy with 5% sodium chloride eye drops administered four times daily and hypertonic ointment applied at night, as well as adjunctive sorbitol-containing lubricating drops. A bandage contact lens had also been attempted; however, it was poorly tolerated, and the approach was discontinued. Despite these measures, no clinically meaningful improvement in corneal clarity or visual acuity was achieved.

### 2.4. Preoperative Assessment and Therapeutic Decision

Given the clinical presentation of complete corneal opacity, the patient had previously been considered unsuitable for EK because of presumed irreversible stromal involvement. PK was considered as an alternative surgical option because of the uncertainty regarding stromal involvement. However, due to systemic comorbidities, increased perioperative risk (ASA III), and the greater burden associated with an open-sky procedure, this approach was considered less favorable.

Considering the AS-OCT findings of marked epithelial thickening and surface irregularity, as well as the possibility that the observed opacity might be at least partially attributable to secondary epithelial pathology rather than definite irreversible stromal scarring, a staged intraoperative strategy was planned. Mechanical removal of the pathological epithelium was proposed as an initial step to enable direct assessment of stromal transparency. If sufficient clarity and visualization of the anterior chamber were achieved, endothelial keratoplasty would be performed during the same procedure.

The choice of DSAEK over DMEK was based on anticipated technical challenges. Significant preoperative corneal thickening and limited visualization raised concerns regarding safe manipulation within the anterior chamber. Under these specific surgical conditions, DSAEK was considered a technically more feasible approach because of the severe corneal edema and limited visualization. The choice of procedure was therefore based on individualized intraoperative and anatomical considerations rather than a general preference for one endothelial keratoplasty technique over another.

### 2.5. Surgical Procedure

**Figure 3:**
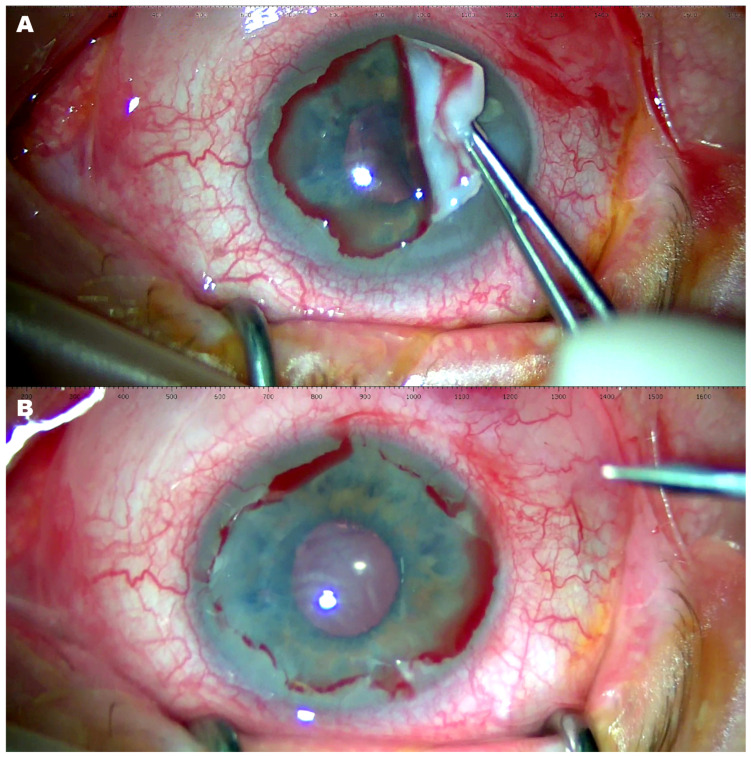
Intraoperative photographs illustrating mechanical debridement of thickened corneal epithelium. (**A**) Removal of the dense, plaque-like epithelial layer using microsurgical forceps, with the underlying corneal surface not yet fully visible. (**B**) Appearance after complete epithelial removal, revealing an edematous but relatively transparent corneal stroma, enabling visualization of anterior chamber structures and facilitating subsequent endothelial keratoplasty.

Surgery was performed under sterile operating conditions. The initial step consisted of mechanical debridement of the pathological thickened epithelium. Using microsurgical forceps, the thickened epithelial plaque was carefully grasped and dissected from the underlying central corneal surface without limbal manipulation (Figure 3A). The pathological epithelium formed a dense, adherent, crust-like structure that was removed in a controlled manner. The removed tissue was submitted for histopathological examination, which demonstrated reactive thickening of the stratified squamous corneal epithelium with associated subepithelial and anterior stromal fibrosis, consistent with chronic bullous keratopathy.

Following complete epithelial removal, partial restoration of corneal transparency was achieved, allowing intraoperative visualization of an edematous but structurally preserved corneal stroma (Figure 3B). This intraoperative reassessment demonstrated that a substantial component of the apparent full-thickness opacity was related to the overlying epithelial pathology. The underlying stromal surface appeared smooth and free of significant scarring, suggesting preserved corneal architecture despite advanced clinical presentation.

Given the improved visualization and preserved stromal architecture after epithelial removal, descemetorhexis was performed and DSAEK was subsequently carried out during the same procedure. An 8.5 mm donor graft with an approximate thickness of 100 μm was prepared using an automated Moria microkeratome system. The graft was inserted into the anterior chamber using a Busin glide with forceps-assisted pull-through technique and successfully positioned on the posterior corneal surface.

To promote graft adherence, a gas tamponade was applied using approximately 30% sulfur hexafluoride (SF_6_) mixed with air. The anterior chamber was initially filled completely to ensure adequate graft apposition to the host stroma. After approximately one hour, partial gas release was performed, leaving the anterior chamber filled to approximately 50% in order to reduce the risk of pupillary block while maintaining sufficient support for graft attachment. Postoperatively, the patient was instructed to maintain a strict supine position during the first 24 h after surgery to support graft adherence. Peripheral iridotomy or surgical iridectomy was not performed.

### 2.6. Postoperative Course and Imaging

**Figure 4:**
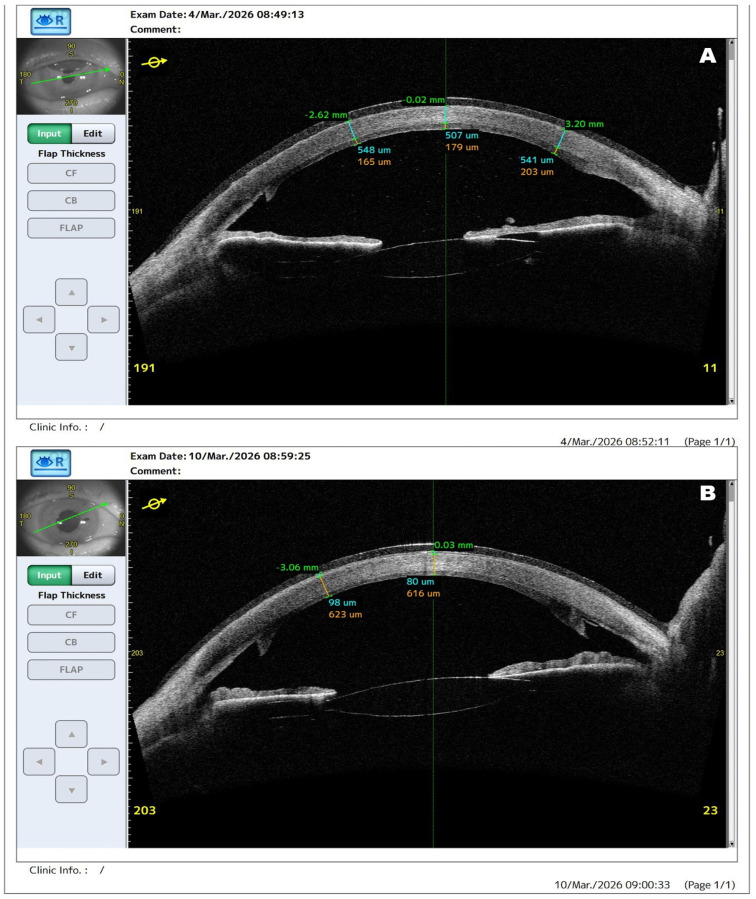
AS-OCT images demonstrating graft attachment and postoperative corneal recovery after DSAEK. (**A**) Early postoperative scan showing a well-positioned posterior lamellar graft fully attached to the host cornea, with visible graft–host interface and no interface fluid. Residual stromal edema is still present in the early postoperative period. (**B**) Follow-up scan confirming stable graft attachment, reduction of stromal edema, improved corneal contour, and restoration of a smoother anterior corneal surface.

Postoperative AS-OCT performed on the first day after surgery demonstrated a well-positioned posterior lamellar graft adherent to the host cornea, with no evidence of interface fluid or graft detachment (Figure 4A). The anterior chamber was well formed, and the graft–host interface appeared optically clear.

Follow-up imaging at one week confirmed stable graft attachment and progressive reduction of stromal edema (Figure 4B). Corneal thickness measured approximately 680 μm on the first postoperative day and gradually decreased to approximately 620 μm at the 6-month follow-up visit. The overall corneal contour also became progressively more regular during follow-up. The anterior corneal surface showed restoration of a continuous and smooth epithelial layer, indicating successful re-epithelialization after mechanical debridement. No signs of epithelial defects or recurrent bullae formation were observed during follow-up.

Postoperatively, the patient was treated with intensive topical therapy, including a fluoroquinolone antibiotic administered six times daily to prevent secondary infection, and topical dexamethasone eye drops administered six times daily. The corticosteroid therapy was gradually tapered over subsequent months according to the postoperative clinical course and graft status. In addition, hyperosmotic eye drops (5% sodium chloride) were continued five times daily to facilitate further reduction of residual corneal edema. Lubricating drops were also used to support epithelial healing and improve ocular surface stability.

IOP remained stable throughout the follow-up period at approximately 15–16 mmHg, with no signs of pressure elevation or pupillary block. The postoperative course was uneventful, with no evidence of early complications such as graft dislocation, significant inflammation, or infection. The prescribed postoperative topical treatment was well tolerated, and no treatment-limiting adverse effects were reported.

### 2.7. Final Outcome

**Figure 5:**
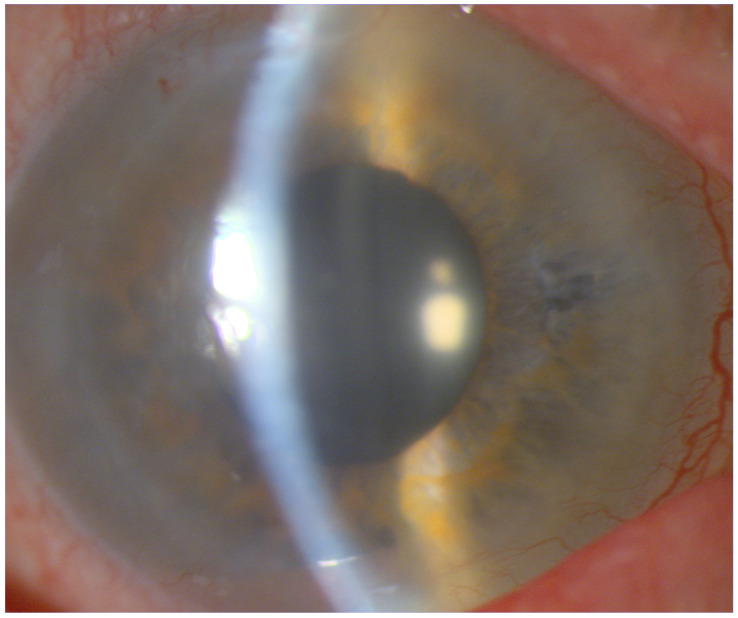
Slit-lamp image showing a clear cornea with restored transparency and a smooth epithelial surface after epithelial debridement and DSAEK, allowing for visualization of anterior chamber structures.

At follow-up, slit-lamp examination demonstrated a clear and transparent cornea with a smooth and stable epithelial surface (Figure 5). The previously observed hyperkeratotic epithelial plaque had completely resolved, and anterior chamber structures were fully visible.

During scheduled postoperative follow-up visits, BCVA improved from hand motion preoperatively to 0.7 Snellen (0.15 logMAR). and remained stable throughout the entire observation period. At 6 months after surgery, endothelial cell density was approximately 1900 cells/mm^2^. Postoperative posterior segment evaluation, including ophthalmoscopy and angio-OCT of the macula, revealed no clinically significant abnormalities limiting visual potential.

At the final postoperative assessment, the graft remained clear, centrally positioned, and fully attached, with no evidence of graft rejection, graft detachment, or need for re-intervention. Residual stromal edema observed during the early postoperative period gradually resolved over subsequent weeks, and no recurrent epithelial abnormalities or recurrent bullae formation were observed during follow-up. The patient continues routine ophthalmological surveillance.

### 2.8. Case Summary

This case illustrates that severe corneal opacity in advanced bullous keratopathy may be predominantly driven by secondary epithelial remodeling rather than irreversible stromal damage. Mechanical removal of the pathological epithelium enabled accurate intraoperative assessment and allowed successful endothelial keratoplasty, resulting in substantial functional visual improvement. The patient also reported marked relief of chronic ocular discomfort and significant improvement in daily functioning after treatment.

## 3. Discussion

### 3.1. Pathophysiology of Bullous Keratopathy and Epithelial Remodeling

Bullous keratopathy represents a consequence of corneal endothelial failure, leading to chronic stromal edema and secondary epithelial alterations. Loss of endothelial pump function results in persistent corneal hydration, disruption of stromal architecture, and progressive epithelial dysfunction manifested by bullae formation, recurrent erosions, and visual impairment [1,2].

Chronic epithelial edema may lead to significant structural remodeling of the corneal surface, including epithelial thickening, irregular stratification, and, in advanced cases, hyperkeratotic transformation. Although such changes are typically considered secondary, their contribution to the overall loss of corneal transparency may be underrecognized in routine clinical assessment. Alterations in epithelial thickness and surface regularity have been shown to influence corneal optics, light scattering, and postoperative visual outcomes following endothelial keratoplasty [3,12].

In the present case, extensive pathological epithelial remodeling created the appearance of complete corneal opacity, effectively masking the underlying stromal condition and complicating preoperative assessment. This observation illustrates that the clinical appearance of advanced endothelial disease may not always accurately reflect the underlying corneal structure.

### 3.2. Diagnostic and Therapeutic Considerations in Surgical Decision-Making

The key clinical challenge in this case was the differentiation between superficial and stromal causes of corneal opacity and the resulting choice of surgical technique. In standard clinical practice, dense corneal opacification is frequently interpreted as a sign of irreversible stromal damage, leading to qualification for penetrating keratoplasty [5,7,11].

However, such an approach may overlook the contribution of epithelial pathology. In this patient, the inability to visualize the anterior chamber and assess stromal clarity preoperatively introduced significant uncertainty. There was a substantial intraoperative risk that, even after removal of the pathological epithelium, the cornea would remain opaque, necessitating conversion to penetrating keratoplasty.

The staged strategy adopted in this case—mechanical epithelial debridement followed by intraoperative reassessment—facilitated stepwise surgical decision-making under conditions of substantial preoperative uncertainty. This approach preserved the possibility of performing EK while maintaining readiness for full-thickness transplantation if required. Superficial keratectomy and epithelial debridement are established techniques for restoring corneal surface regularity and may also serve as diagnostic tools in complex cases [13].

From a therapeutic perspective, EK has become the standard of care for corneal endothelial failure, largely replacing PK due to its improved safety profile and functional outcomes [5,6]. Numerous studies have demonstrated that endothelial keratoplasty is associated with faster visual rehabilitation, improved refractive stability, and lower complication rates compared with penetrating keratoplasty [4,9,10]. Previous comparative studies have also reported favorable visual and optical outcomes following lamellar keratoplasty techniques [8,10,14,15,16,17].

Importantly, PK is associated with a higher risk of immunological graft rejection, often necessitating prolonged and intensive corticosteroid therapy, and in selected cases, systemic immunosuppression [18,19]. In contrast, EK demonstrates significantly lower rejection rates, allowing postoperative management primarily with topical therapy [6,18]. This distinction is particularly relevant in patients with systemic comorbidities, in whom systemic treatment may increase the risk of adverse events.

In eyes with compromised visualization, DSAEK may provide technical advantages related to graft manipulation and intraoperative handling. Graft thickness predictability and surgical reproducibility contribute to stable anatomical outcomes [20,21]. In the present case, increased corneal thickness and limited intraoperative visibility reduced the feasibility of DMEK, which requires precise manipulation of a thin and fragile graft [22,23]. Therefore, in the present case, DSAEK was considered the most technically feasible EK technique under the intraoperative conditions encountered.

Furthermore, the overall postoperative burden associated with penetrating keratoplasty—including longer recovery time, higher complication rates, and the need for suture management—may significantly impact patient quality of life. In the present case, successful qualification for DSAEK allowed avoidance of these limitations, providing a safer and less burdensome therapeutic alternative.

### 3.3. Postoperative Considerations and Functional Outcome

An important clinical advantage of EK is the reduced risk of immunological graft rejection compared with PK. While this difference has been well established in the literature [6,18,19], its relevance is particularly pronounced in patients with systemic comorbidities.

In the present case, the patient’s diabetes mellitus and chronic heart failure increased the potential risks associated with prolonged or systemic immunosuppressive therapy. Therefore, the ability to manage the postoperative course using topical corticosteroids alone represented a significant therapeutic advantage.

In the present case, the lower immunological burden and reduced surgical invasiveness were additional factors supporting the choice of a lamellar approach. Visual recovery in the present case was favorable, with improvement from hand motion preoperatively to 0.15 logMAR at follow-up, accompanied by restoration of corneal transparency and a stable epithelial surface. Although endothelial keratoplasty is generally associated with good functional outcomes [10,24,25,26,27], the postoperative result observed in this patient should be interpreted cautiously given the single-case nature of the report.

### 3.4. Clinical Implications

The present case illustrates an important diagnostic pitfall in the evaluation of advanced bullous keratopathy, where the clinical appearance of diffuse corneal opacity may not accurately reflect the extent of irreversible stromal damage.

This observation has direct implications for surgical planning, as reliance on slit-lamp findings alone may lead to overestimation of disease severity and inappropriate qualification for PK. In such cases, failure to consider the contribution of reversible surface pathology may result in unnecessarily invasive treatment.

From a practical perspective, this case suggests that targeted surface assessment strategies, including mechanical epithelial debridement, may facilitate intraoperative evaluation of corneal transparency in carefully selected patients when preoperative assessment remains inconclusive.

Although no general conclusions can be drawn from a single case, the present observation supports a cautious and individualized approach to surgical decision-making in complex cases of bullous keratopathy.

### 3.5. Limitations

This report describes a single clinical case, and the findings should be interpreted with caution. Although histopathological examination confirmed reactive epithelial thickening and associated fibrosis, the precise contribution of individual epithelial and stromal components to the observed loss of corneal transparency could not be quantitatively determined.

In addition, reliable preoperative endothelial assessment in the affected eye was not possible because of the severe corneal opacity and epithelial surface irregularity. The conclusion regarding preservation of stromal architecture was therefore based primarily on intraoperative reassessment after epithelial removal.

The favorable postoperative outcome observed in this patient may also reflect a highly selected anatomical and surgical situation and should not be interpreted as broadly generalizable to all cases of advanced bullous keratopathy with diffuse corneal opacity. Furthermore, the decision to proceed with DSAEK was made intraoperatively and depended on the improved visualization achieved after epithelial debridement, as well as surgeon experience and available surgical alternatives.

Finally, the follow-up period remains relatively limited, and further studies involving larger patient cohorts are needed to better define the prevalence and clinical relevance of this phenomenon and to establish more standardized approaches to surgical decision-making in similar cases.

### 3.6. Discussion Summary

This case highlights the diagnostic difficulty of assessing corneal transparency in advanced bullous keratopathy when severe epithelial remodeling obscures deeper corneal structures. Mechanical epithelial debridement enabled intraoperative reassessment of stromal clarity and facilitated individualized surgical decision-making. The favorable postoperative outcome observed in this patient suggests that careful evaluation of epithelial contributions to apparent corneal opacity may be clinically relevant in selected cases.

## 4. Conclusions

This case illustrates that apparent full-thickness corneal opacity in advanced bullous keratopathy may, in selected patients, be substantially influenced by reversible epithelial pathology rather than solely by irreversible stromal damage. Advanced epithelial remodeling may obscure the true structural condition of the cornea and lead to misinterpretation of surgical eligibility.

Mechanical removal of the pathological epithelium served as a critical diagnostic and therapeutic step, enabling accurate intraoperative assessment of stromal transparency. In the present case, this approach enabled successful qualification for EK and avoidance of PK, which was considered less favorable because of the patient’s clinical and systemic characteristics.

The present case also suggests the potential value of a staged surgical strategy incorporating epithelial debridement and intraoperative reassessment in selected patients with inconclusive preoperative evaluation. Such an approach may be especially relevant in patients with systemic comorbidities, where minimizing surgical invasiveness and treatment burden is essential.

Overall, this case highlights the importance of considering epithelial contributions to corneal opacity and supports cautious intraoperative reassessment in selected patients with uncertain preoperative evaluation.

## Figures and Tables

**Table 1:** Chronological timeline of the patient’s clinical course, diagnostic assessment, treatment, and follow-up.

Time Point	Clinical Course
Approximately 10 months before surgery	Progressive visual deterioration and ocular discomfort developed in the right eye.
Preoperative period	Conservative treatment with hyperosmotic drops, ointment, lubricants, and a bandage contact lens trial did not improve corneal clarity or visual acuity.
Before surgical qualification	The patient initially declined surgery; penetrating keratoplasty was considered high risk because of systemic comorbidities.
Qualification visit	Slit-lamp examination showed complete corneal opacity; AS-OCT revealed marked epithelial thickening, irregularity, and epithelial bullae; B-scan showed no significant posterior segment abnormalities.
Day of surgery	Mechanical epithelial debridement was performed, followed by intraoperative reassessment and DSAEK.
Postoperative day 1	AS-OCT confirmed graft attachment without interface fluid or detachment.
Postoperative week 1	Stable graft attachment, reduced stromal edema, and complete re-epithelialization were observed.
At least 6 months after surgery	BCVA remained stable at 0.7 Snellen (0.15 logMAR), with a clear cornea and no graft-related complications.

## Data Availability

The original contributions presented in this study are included in the article. Further inquiries can be directed to the corresponding author.

## Abbreviations

The following abbreviations are used in this manuscript:

ASA	American Society of Anesthesiologists
AS-OCT	Anterior Segment Optical Coherence Tomography
BCVA	Best-corrected Visual Acuity
DMEK	Descemet membrane endothelial keratoplasty
DSAEK	Descemet Stripping Automated Endothelial Keratoplasty
EK	Endothelial keratoplasty
IOP	Intraocular pressure
PK	Penetrating keratoplasty

## References

[1] Chang V.S., Gibbons A., Osigian C. (2020). Phacoemulsification in the Setting of Corneal Endotheliopathies: A Review. Int. Ophthalmol. Clin..

[2] Deguchi H., Tanioka H., Watanabe M., Horiuchi N., Fukuoka H., Hieda O., Inatomi T., Kinoshita S., Sotozono C. (2024). Identification and Analysis of Primary Cilia in the Corneal Endothelial Cells of Patients with Bullous Keratopathy. Curr. Eye Res..

[3] Nielsen E., Ivarsen A., Kristensen S., Hjortdal J. (2016). Fuchs’ Endothelial Corneal Dystrophy: A Controlled Prospective Study on Visual Recovery After Endothelial Keratoplasty. Acta Ophthalmol..

[4] Price M.O., Giebel A.W., Fairchild K.M., Price F.W. (2009). Descemet’s Membrane Endothelial Keratoplasty: Prospective Multicenter Study of Visual and Refractive Outcomes and Endothelial Survival. Ophthalmology.

[5] Stuart A.J., Romano V., Virgili G., Shortt A.J. (2018). Descemet’s Membrane Endothelial Keratoplasty (DMEK) versus Descemet’s Stripping Automated Endothelial Keratoplasty (DSAEK) for Corneal Endothelial Failure. Cochrane Database Syst. Rev..

[6] Deng S.X., Lee W.B., Hammersmith K.M., Kuo A.N., Li J.Y., Shen J.F., Weikert M.P., Shtein R.M. (2018). Descemet Membrane Endothelial Keratoplasty: Safety and Outcomes: A Report by the American Academy of Ophthalmology. Ophthalmology.

[7] Woo J.H., Ang M., Htoon H.M., Tan D. (2019). Descemet Membrane Endothelial Keratoplasty Versus Descemet Stripping Automated Endothelial Keratoplasty and Penetrating Keratoplasty. Am. J. Ophthalmol..

[8] Maier A.B., Milek J., Joussen A.M., Dietrich-Ntoukas T., Lichtner G. (2023). Systematic Review and Meta-Analysis: Outcomes After Descemet Membrane Endothelial Keratoplasty Versus Ultrathin Descemet Stripping Automated Endothelial Keratoplasty. Am. J. Ophthalmol..

[9] van Dijk K., Ham L., Tse W.H., Liarakos V.S., Quilendrino R., Yeh R.Y., Melles G.R. (2013). Near Complete Visual Recovery and Refractive Stability in Modern Corneal Transplantation: Descemet Membrane Endothelial Keratoplasty (DMEK). Cont. Lens Anterior Eye.

[10] Dunker S.L., Dickman M.M., Wisse R.P.L., Nobacht S., Wijdh R.H.J., Bartels M.C., Tang M.L., van den Biggelaar F.J.H.M., Kruit P.J., Nuijts R.M.M.A. (2020). Descemet Membrane Endothelial Keratoplasty versus Ultrathin Descemet Stripping Automated Endothelial Keratoplasty: A Multicenter Randomized Controlled Clinical Trial. Ophthalmology.

[11] Castellucci M., Novara C., Casuccio A., Cillino G., Giordano C., Failla V., Bonfiglio V., Vadalà M., Cillino S. (2021). Bilateral Ultrathin Descemet’s Stripping Automated Endothelial Keratoplasty vs. Bilateral Penetrating Keratoplasty in Fuchs’ Dystrophy: Corneal Higher-Order Aberrations, Contrast Sensitivity and Quality of Life. Medicina.

[12] Levy I., Habib L., Morgan S., Mukhija R., Nanavaty M.A. (2026). Epithelial Thickness Changes After Descemet Membrane Endothelial Keratoplasty (DMEK): An Observational Study. J. Clin. Med..

[13] Salari F., Beikmarzehei A., Liu G., Zarei-Ghanavati M., Liu C. (2022). Superficial Keratectomy: A Review of Literature. Front. Med..

[14] Duggan M.J., Rose-Nussbaumer J., Lin C.C., Austin A., Labadzinzki P.C., Chamberlain W.D. (2019). Corneal Higher-Order Aberrations in Descemet Membrane Endothelial Keratoplasty Versus Ultrathin DSAEK in the Descemet Endothelial Thickness Comparison Trial: A Randomized Clinical Trial. Ophthalmology.

[15] Dimtsas G.S., Moschos M.M. (2023). Ultrathin-Descemet Stripping Automated Endothelial Keratoplasty Versus Descemet Membrane Endothelial Keratoplasty: A Systematic Review and Meta-Analysis. In Vivo.

[16] Sela T.C., Iflah M., Muhsen K., Zahavi A. (2023). Descemet Membrane Endothelial Keratoplasty Compared with Ultrathin Descemet Stripping Automated Endothelial Keratoplasty: A Meta-Analysis. BMJ Open Ophthalmol..

[17] Lin C.C., Chamberlain W.D., Kakigi C., Arnold B.F., Rose-Nussbaumer J. (2024). Mediators of Visual Acuity in Descemet Membrane Endothelial Keratoplasty and Ultrathin Descemet Stripping Automated Endothelial Keratoplasty. Cornea.

[18] Anshu A., Price M.O., Price F.W. (2012). Risk of Corneal Transplant Rejection Significantly Reduced with Descemet’s Membrane Endothelial Keratoplasty. Ophthalmology.

[19] Hos D., Tuac O., Schaub F., Stanzel T.P., Schrittenlocher S., Hellmich M., Bachmann B.O., Cursiefen C. (2017). Incidence and Clinical Course of Immune Reactions after Descemet Membrane Endothelial Keratoplasty: Retrospective Analysis of 1000 Consecutive Eyes. Ophthalmology.

[20] Nishisako S., Yamaguchi T., Kusano Y., Higa K., Aoki D., Sasaki C., Shimazaki J. (2022). The Predictability of Graft Thickness for Descemet’s Stripping Automated Endothelial Keratoplasty Using a Mechanical Microkeratome System. Sci. Rep..

[21] Geber A., Masnec S., Kalauz M., Bešlić I., Škegro I., Gaćina D.L., Jandroković S., Meter A., Kuzman T. (2025). Comparative Analysis of Corneal Morphological and Optical Parameters in Predicting DSAEK Surgery Outcome. Medicina.

[22] Colby K. (2024). Cornea Classics: Melles, Ong, Ververs, and van der Wees, “Descemet Membrane Endothelial Keratoplasty (DMEK)” (2006). Cornea.

[23] Tourtas T., Laaser K., Bachmann B.O., Cursiefen C., Kruse F.E. (2012). Descemet Membrane Endothelial Keratoplasty Versus Descemet Stripping Automated Endothelial Keratoplasty. Am. J. Ophthalmol..

[24] Monnereau C., Quilendrino R., Dapena I., Liarakos V.S., Alfonso J.F., Arnalich-Montiel F., Böhnke M., Pereira N.C., Dirisamer M., Parker J. (2014). Multicenter Study of Descemet Membrane Endothelial Keratoplasty: First Case Series of 18 Surgeons. JAMA Ophthalmol..

[25] Terry M.A., Shamie N., Chen E.S., Phillips P.M., Shah A.K., Hoar K.L., Friend D.J. (2009). Endothelial Keratoplasty for Fuchs’ Dystrophy with Cataract: Complications and Clinical Results with the New Triple Procedure. Ophthalmology.

[26] Marques R.E., Guerra P.S., Sousa D.C., Gonçalves A.I., Quintas A.M., Rodrigues W. (2019). DMEK versus DSAEK for Fuchs’ Endothelial Dystrophy: A Meta-Analysis. Eur. J. Ophthalmol..

[27] Price F.W., Price M.O. (2005). Descemet’s Stripping with Endothelial Keratoplasty in 50 Eyes: A Refractive Neutral Corneal Transplant. J. Refract. Surg..

